# Simvastatin Results in a Dose-Dependent Toxic Effect on Spiral Ganglion Neurons in an* In Vitro* Organotypic Culture Assay

**DOI:** 10.1155/2016/3580359

**Published:** 2016-03-14

**Authors:** Katharina Leitmeyer, Andrea Glutz, Cristian Setz, Leonie Wieland, Sulamith Egloff, Daniel Bodmer, Yves Brand

**Affiliations:** ^1^Department of Biomedicine, University Hospital Basel, Hebelstrasse 20, 4031 Basel, Switzerland; ^2^Clinic for Otolaryngology, Head and Neck Surgery, University Hospital Basel, Petersgraben 4, 4031 Basel, Switzerland

## Abstract

Statins are inhibitors of the 3-hydroxy-3-methylglutaryl-coenzyme A reductase, an enzyme necessary for the production of mevalonate. They are widely used as cholesterol-lowering drugs. However, conflicting data about the effect of statins on neuronal cells has been published. To explore the effect of simvastatin on spiral ganglion neurons (SGNs), SG explants of 5-day-old rats were treated with increasing concentrations of simvastatin. In addition, SG explants were treated with mevalonate and with the combination of simvastatin and mevalonate. SGN number, length of the neurites, area of nonneuronal supporting cells, and neuronal survival were analyzed. Simvastatin treatment results in a significant dose-dependent decrease of SG neurite number, length of neurites, area of supporting cells, and SG neuronal survival compared to control. Interestingly, treatment with mevalonate in addition to simvastatin increased SG neuronal survival compared to simvastatin treatment only. However, treatment with mevalonate in addition to simvastatin did not influence SG neurite number, length of neurites, and area of supporting cells compared to simvastatin treatment only. Our results suggest a neurotoxic effect of simvastatin on SGNs* in vitro*. Neurotoxicity seems to be at least partially mediated by the mevalonate pathway. Therefore, caution is warranted to use simvastatin as a potential otoprotective drug.

## 1. Introduction

Sensorineural hearing loss is linked to degeneration and death of auditory hair cells (HCs) and their associated spiral ganglion neurons (SGNs), which is irreversible in mammals. Despite the progress made towards understanding the processes involved in HC and SGN death and survival, there is still no available cure for individuals with sensorineural hearing loss; only auditory prosthesis (e.g., hearing aids or cochlear implants) can offer some help to individuals with hearing loss. Therefore, developing therapeutic strategies for hearing loss prevention is one of the major goals of current auditory research. Among the potential otoprotective drugs are statins.

Statins are inhibitors of 3-hydroxy-3-methylglutaryl-coenzyme A (HMG-CoA) reductase and commonly used for the treatment of hyperlipidemia [1]. The HMG-CoA reductase is a rate-limiting enzyme in the cholesterol biosynthesis. Inhibition of HMG-CoA reductase results in a reduction of cholesterol in plasma and an increased expression of low-density lipoprotein (LDL) receptors [2]. Statins reduce the incidence of primary and secondary coronary heart disease in clinic trials and act by blocking the enzyme necessary for the production of L-mevalonate, an intermediary product in the synthesis of cholesterol [3, 4]. Moreover, statins also reduce the risk of atherosclerosis and have anti-inflammatory, immunomodulatory, and apoptotic effects [5].

During the past decade, statin treatment has been discussed to prevent or improve sensorineural hearing loss [6, 7]. Some clinical studies suggest that diet control and antilipid therapy improve sensorineural hearing loss associated with hyperlipidemia [8–10]. However, Olzowy et al. [11] did not find an effect of atorvastatin on progression of sensorineural hearing loss in the elderly in a prospective, randomized, double-blinded clinical trial. Interestingly, Chung et al. [12] showed an association between previous statin use and sudden sensorineural hearing loss. It has been demonstrated that hyperlipidemia and atherosclerosis in apolipoprotein E knockout (ApoE-KO) mice resulted in structural and functional changes in the inner ear, which were associated with hearing loss in a time-dependent manner [13, 14]. Cai et al. [15] found that simvastatin treatment protects the hearing of ApoE-KO mice that were fed a high fat diet. They attribute these results to reduced atherosclerotic lesions and control of hyperlipidemia. Syka et al. [16] found a protective effect of atorvastatin on the inner ear. They explain their results by reduction of endothelial inflammatory effects which influence the blood supply to the inner ear. Our group recently demonstrated that simvastatin protects HCs from gentamicin-induced toxicity* in vitro* [17]. However, the effects of simvastatin have not yet been investigated on SGNs.

Given these conflicting data, we examined the effect of statins on SGNs* in vitro*. The aim of the present study was to evaluate the effect of simvastatin on SG neuronal survival, neuritogenesis, and neurite elongation* in vitro*.

## 2. Material and Methods

### 2.1. Preparation of Tissue Culture Plates

All experiments were performed as previously described by our group [18–23]. First, uniformly coated 24-well cell culture plates (Costar®, Corning Inc., Acton, MA, USA) were prepared and the wells were filled with 300 *μ*L of 5 *μ*g/mL poly-L-lysine (PLL) (Sigma-Aldrich, St. Louis, USA) in Dulbecco's modified Eagle's medium (DMEM) (Gibco by Invitrogen, Carlsbad, USA). Next, the culture plates were incubated at 37°C for 1 hour. The wells were then washed twice with phosphate buffered saline (PBS) and filled with 170 *μ*L of primary attachment medium, containing DMEM (Gibco), 10% fetal bovine serum (Sigma-Aldrich), 25 mM HEPES buffer (Gibco), and 300 U/mL penicillin (Sigma-Aldrich).

### 2.2. SG Dissection

All animal procedures were carried out according to an approved animal research protocol (Kantonales Veterinaeramt, Basel, Switzerland). Neonatal 5-day-old Wistar rats (Harlan, Netherlands) were euthanized. The cochlea and the spiral ganglion were removed and further dissected similar to the method described by Van de Water and Ruben [24]. Briefly, the cochlear capsule was opened and the membranous labyrinth was removed from the modiolus. The spiral lamina containing the SG was carefully separated from the modiolus and transferred immediately into primary cell culture medium, where it was then cut into equal portions of 300 *μ*m to 500 *μ*m before being transferred to the prepared culture plates. Each explant was cultured in a separate culture well.

### 2.3. Cell Culture

First, explants were incubated for 24 h at 37°C in primary attachment medium, and the culture medium was subsequently changed to serum-free maintenance medium (DMEM (Gibco), 25 mM HEPES buffer (Gibco), 6 mg/mL glucose (Gibco), 300 U/mL penicillin (Sigma-Aldrich), and 30 *μ*L/mL N2-supplement (Gibco)). Maintenance medium was supplemented with 10 ng/mL of recombinant BDNF for trophic support of SGN survival and optimization of neurite outgrowth (R&D Systems, Minneapolis, MN, USA). Cultures were kept in a humidified incubator at 5% CO_2_ and 37°C for 72 h. Experimental cultures received various concentrations (1 *μ*M, 10 *μ*M, or 100 *μ*M, resp.) of simvastatin (Sigma-Aldrich), mevalonate (10 *μ*M, Sigma-Aldrich), or simvastatin and mevalonate (both 10 *μ*M, Sigma-Aldrich). Culture media with DMSO only served as control. Simvastatin was dissolved in DMSO and the same DSMO concentration as in the samples treated with 100 *μ*M simvastatin was used. Simvastatin was converted into the active acid following the protocol of Bogman et al. [25] prior to its use. Stock solutions of 10 *μ*M simvastatin in DMSO were stored at −20°C. 20 SG explants were analyzed per experimental condition.

### 2.4. Immunohistochemistry

First, the explants were fixed with 4% paraformaldehyde for 20 min at room temperature (RT). Then, the explants were washed twice with PBS (Gibco) and permeabilized with 5% triton X-100 (Sigma-Aldrich) for 10 min. After permeabilization, the explants were again washed twice with PBS (Gibco) and then blocked for nonspecific antibody binding with 5% donkey serum (Sigma-Aldrich). Neurites were labeled for neurofilament using a mouse polyclonal 200 kDa anti-neurofilament primary antibody (1 : 400; Sigma-Aldrich). After primary antibody incubation overnight at 4°C, followed by two PBS washes, the neurites were visualized by 2.5 h of incubation with fluorescein isothiocyanate (FITC) conjugated secondary antibodies (1 : 100; Jackson Immunoresearch, West Grove, PA, USA) against mouse antibody.

### 2.5. Quantification of Neuronal Survival

To assess effects on neuronal survival, half turn SG explants were cultured as above for 72 hours, except that the explants were grown on glass cover slips. The explants were fixed as above, treated with 0.5% peroxide in methanol to block endogenous peroxidases, reacted with a mouse monoclonal antibody IgG against rat neurofilament 200 (Sigma-Aldrich), followed by a biotinylated secondary anti-mouse IgG, and developed by an avidin and DAB procedure (Vector Laboratories, Burlingame, CA). The tissue was cleared with citrosol (Fischer Scientific, Waltham, MA, USA) to allow visualization of the cell somas for evaluation of neuronal survival. 12 SG explants were studied per condition.

### 2.6. Data Analysis

Digital images for immunohistochemistry were obtained on a fluorescence microscope (Olympus IX71, Center Valley, PA, USA) and photographed with an AxioCam (Zeiss, San Diego, USA). Digital images for quantification of neuronal survival were obtained on an inverted microscope (Olympus BX63 Center Valley, PA, USA). For publication in this paper, images were optimized to achieve uniform brightness and contrast using Adobe Photoshop (Adobe Systems Inc., San Jose, CA, USA). Neurite outgrowth from the SG was evaluated by measuring the number and lengths of the processes. Growth of supporting cells was evaluated by measuring the area of the skirt surrounding the SGN. Images of the immunostained cultures were analyzed by using ImageJ software (NIH, Bethesda, MD, USA). Each neurite was traced and number of neurites, average lengths of neurites, and area of the supporting cells per explant were analyzed. Neuronal survival was analyzed by evaluating the number of neurons per 100 *μ*m. A viable neuron fulfilled the following criteria: cell bodies with an intact cell membrane, no evidence of DNA fragmentation, and ultrastructurally homogeneous cytosol. Neurons with signs of apoptosis (DNA-fragmented nucleus, condensed chromatin, and membrane boiling or blebs or apoptotic bodies) were excluded [26, 27]. Statistical analysis was performed using a one-way analysis of variance (ANOVA) followed by Tukey least-significant-difference post hoc test with Bonferroni correction. Data presented in the text and figures are means and standard deviations. Results were considered to be significant when the likelihood for a type 1 error was less than 5% (*p* < 0.05).

## 3. Results

### 3.1. Treatment with Simvastatin Results in a Dose-Dependent Decrease in SG Neurite Number

**Figure 1:**
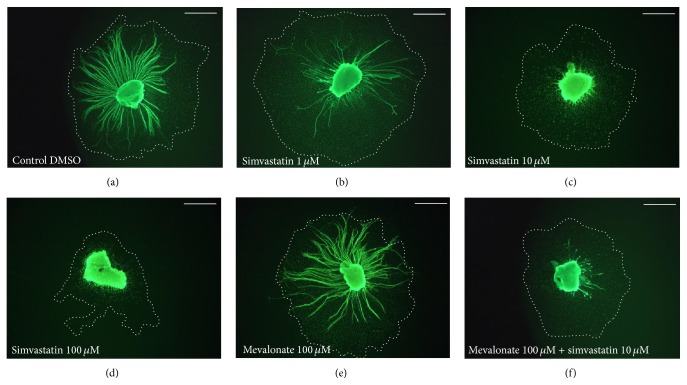
*Representative examples of rat SG explants stained with anti-neurofilament antibody*. (a) Representative example of a rat SG explant grown for 72 h in culture only. (b) Representative example for SG treated for 72 h with simvastatin 1 *μ*M. (c) Representative example for SG treated for 72 h with simvastatin 10 *μ*M. (d) Representative example for SG treated for 72 h with simvastatin 100 *μ*M. (e) Representative example for SG treated for 72 h with mevalonate 100 *μ*M. (f) Representative example for SG treated for 72 h with mevalonate 100 *μ*M and simvastatin 1 *μ*M. Dash lines indicate the area of supporting cells. Scale bar 250 *μ*m.

**Figure 2:**
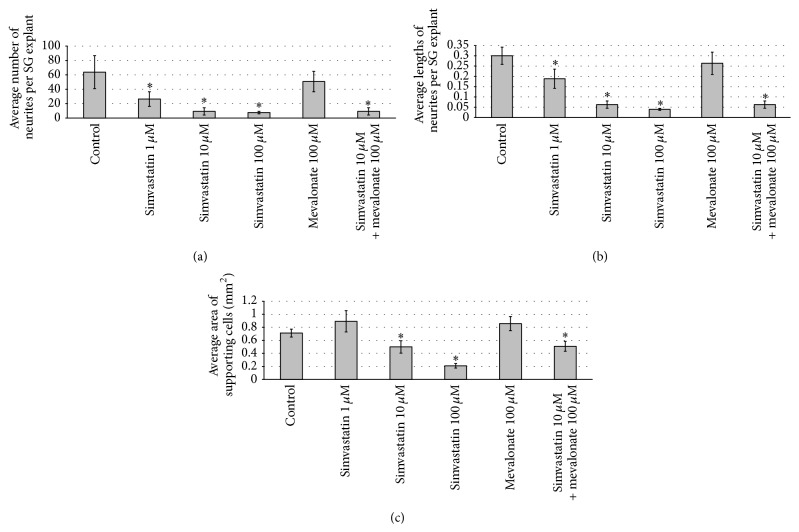
*Quantitative analysis of SG neurites number, SG neurites length, and area of supporting cells*. (a) Effect of simvastatin on the number of neurites from SG explants. There was a statistically significant decrease in the number of neurites in SG treated with simvastatin compared to control (ANOVA, *p* < 0.05). Mevalonate treatment had no effect on the number of neurites compared to control. The combination of simvastatin 10 *μ*M and mevalonate 100 *μ*M resulted in a decrease in the number of neurites per SG explant compared to control (ANOVA, *p* < 0.05). (b) Effect of simvastatin on the length of neurites from SG explants. There was a statistically significant decrease in the length of neurites in SG treated with simvastatin compared to control (ANOVA, *p* < 0.05). Mevalonate treatment had no effect on the length of neurites compared to control. The combination of simvastatin 10 *μ*M and mevalonate 100 *μ*M resulted in a decrease in the length of neurites per SG explant compared to control (ANOVA, *p* < 0.05). (c) Effect of simvastatin on the area of supporting cells. There was a statistically significant decrease in the area of supporting cells in SG treated with simvastatin compared to control (ANOVA, *p* < 0.05). Mevalonate treatment had no effect on the area of supporting cells compared to control. The combination of simvastatin 10 *μ*M and mevalonate 100 *μ*M resulted in a decrease in the area of supporting cells compared to control (ANOVA, *p* < 0.05). *∗* denotes statistical difference compared to control (*p* < 0.05).

Simvastatin treatment results in a decrease in number of neurites per SG explant. The average number of neurites was decreased compared to control in all concentrations of simvastatin used in this study (1 *μ*M, 10 *μ*M, and 100 *μ*M; ANOVA *p* < 0.05 for all conditions). This effect was dose-dependent (Figures 1 and 2).

### 3.2. Treatment with Simvastatin Results in a Dose-Dependent Decrease in Length of SG Neurites

Simvastatin treatment results in a decrease in length of neurites per SG explant. The average length of neurites was decreased compared to control in all concentrations of simvastatin used in this study (1 *μ*M, 10 *μ*M, and 100 *μ*M; ANOVA *p* < 0.05 for all conditions). This effect was dose-dependent (Figures 1 and 2).

### 3.3. Treatment with Simvastatin Results in Decreased Area of Supporting Cells

Simvastatin also significantly decreased the area of nonneuronal cells, which have been previously identified as fibroblasts and Schwann cells [28] growing around the explant, as compared to the negative control. This effect was dose-dependent and significant for the two highest concentrations used in this study (10 *μ*M and 100 *μ*M; ANOVA *p* < 0.05 for both conditions) (Figures 1 and 2).

### 3.4. Simvastatin Decreases SG Neuronal Survival

**Figure 3:**
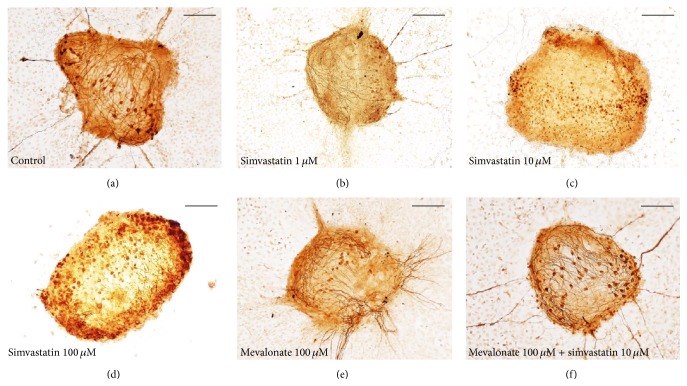
*Representative examples of rat SG explants stained to assess neuronal survival*. (a) Representative example of a rat SG explant grown for 72 h in culture only. (b) Representative example for SG treated for 72 h with simvastatin 1 *μ*M. (c) Representative example for SG treated for 72 h with simvastatin 10 *μ*M. (d) Representative example for SG treated for 72 h with simvastatin 100 *μ*M. (e) Representative example for SG treated for 72 h with mevalonate 100 *μ*M. (f) Representative example for SG treated for 72 h with mevalonate 100 *μ*M and simvastatin 1 *μ*M. Scale bar 150 *μ*m.

**Figure 4:**
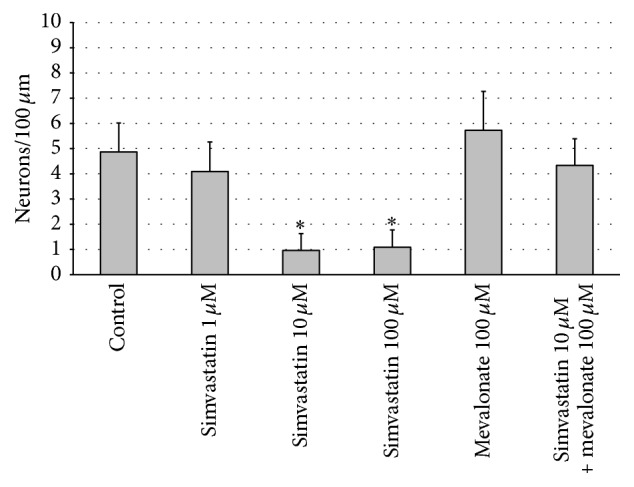
*Quantitative analysis of SG neuronal survival*. Treatment with simvastatin 10 *μ*M and 100 *μ*M resulted in decreased SG neuronal survival compared to control (ANOVA, *p* < 0.05). Treatment with simvastatin 1 *μ*M, mevalonate 100 *μ*M, and the combination of simvastatin 10 *μ*M and mevalonate 100 *μ*M did not influence SG neuronal survival compared to control. *∗* denotes statistical difference compared to control (*p* < 0.05).

The decreased number of neurites extending from SG explants could reflect the altered survival and/or neuritogenesis of SGNs. To assess this, we evaluated the survival of SGN cell bodies within explants. Simvastatin decreased SG neuronal survival when compared to controls in the two highest concentrations used in this study (10 *μ*M and 100 *μ*M; ANOVA, *p* > 0.05 for both conditions) (Figures 3 and 4).

### 3.5. Mevalonate Does Not Affect SG Neurite Number, SG Neurite Length, nor SG Neuronal Survival

Treatment with mevalonate did not influence SG neurite number, SG neurite length, or SG neuronal survival compared to control (Figures 1–4).

### 3.6. Treatment with Mevalonate in addition to Simvastatin Increases SG Neuronal Survival Compared to Simvastatin Treatment

Interestingly, treatment with mevalonate in addition to simvastatin increased SG neuronal survival compared to simvastatin treatment only (ANOVA *p* > 0.05; Figures 3 and 4). However, treatment with mevalonate in addition to simvastatin did not influence SG neurite number, SG neurite length, nor the area of nonneuronal cells around the SG explants compared to simvastatin treatment only (Figures 1 and 2).

## 4. Discussion

During the last decade, it has been hypothesized that statins might have a neuroprotective effect and therefore might be a potential drug for the treatment for sudden sensorineural hearing loss [6]. Recently, our group showed a protective effect of simvastatin on gentamicin-induced HC loss* in vitro*. We proposed that statins act by enhancing Akt activation and decrease the isoprenylation of small G proteins, such as Ras and Rho/Rac/Cdc42 [17]. However, we did not analyze the effect on SGN in our study. Cai et al. [15] discussed that statins prevent hearing loss due to reduction of atherosclerotic lesions and levels of glucose, cholesterol, low-density lipoproteins, and triglyceride. Moreover, Chang et al. [29] described a relationship between hyperlipidemia and hearing problems.

In contrast, Chung et al. [12] showed that sudden sensorineural hearing loss was significantly associated with previous statin use. Moreover, toxic effects of simvastatin are described in the inner ear [5]. The authors found neurodegenerative morphological changes and cell death after simvastatin treatment in cultured cochlear neuronal cells. The authors explain that this could be caused by a reduction of mevalonate pathway products, which are important antioxidants and membrane stabilizers. Simvastatin reduces the production of mevalonate by blocking the enzyme necessary for the production of mevalonate [3, 4].

Given these conflicting data, we evaluate the effects of simvastatin on SG neurites* in vitro*. Our data shows that simvastatin decreases the number of SG neurites, reduces the length of SG neurites, and also decreases the area of nonneuronal supporting cells around the SGNs (Figures 1 and 2). Moreover, we found that simvastatin reduced SG neuronal survival (Figures 3 and 4). Therefore, our results indicate that simvastatin is toxic for SGNs* in vitro*.

How can the toxic effect on SGNs of simvastatin be explained? Statins are inhibitors of the 3-hydroxy-3-methylglutaryl-coenzyme A reductase, an enzyme necessary for the production of mevalonate. Mevalonate is essential for the production of coenzyme Q10 and statins lead to a dose-dependent reduction in Q10 [30]. Q10 is a stabilizer of mitochondrial membranes and has an antiapoptotic effect [31]. Inhibition of the mevalonate pathway could be an explanation of our observations such that addition of mevalonate to simvastatin could rescue the SGN from simvastatin toxicity in our SGN neuronal survival experiments (Figures 3 and 4). We hypothesize that mammalian SGN may be more vulnerable to Q10 reduction by simvastatin than cochlear HCs. However, addition of mevalonate to simvastatin had no influence on SG neurite number, SG neurite length, and the area of nonneuronal cells around the SGNs compared to simvastatin treatment only. This indicates that the effect of simvastatin on SGNs and nonneuronal supporting cells is only partially mediated by the mevalonate pathway and alternative mechanisms mediating the toxic effects of simvastatin have to be considered.

It should be noted that in our experiments we used organotypic explants from the cochlear SG that consisted of neurons and nonneuronal supporting cells, including fibroblasts and Schwann cells. Both cells might have influenced the observed reduction in neuritogenesis and length of neurites in our experiments. Moreover we could not distinguish between the dendrites and axons of SGN because there exist no differentiating markers. Two different subtypes with different functions and cellular interactions of SGNs are known, type I and type II SGNs [32]. The dendrites of type I cells are involved in afferent synapses exclusively with the IHCs, while the dendrites of type II cells exclusively interact with the OHCs [32]. It should be noted that in the present study we could not distinguish between type I and type II SGNs. 95% of SGNs are type I cells; therefore it seems likely that this subtype of neuron dominates our results. We used 5-day-old rat SGNs. In the rat cochlea onset of hearing approximately occurs on postnatal day 10 [33, 34]. We studied prehearing neurons because of the increased difficultly to culture older neurons. In addition, neurite development is still ongoing in 5-day-old rats [35, 36].

In summary, our data indicates a toxic effect of simvastatin on SG neuritogenesis, SG neuronal survival, and nonneuronal supporting cells* in vitro*. Therefore, caution is warranted to use simvastatin as a potential otoprotective drug.

## References

[1] Dirks A. J., Jones K. M. (2006). Statin-induced apoptosis and skeletal myopathy. *The American Journal of Physiology—Cell Physiology*.

[2] Liao J. K., Laufs U. (2005). Pleiotropic effects of statins. *Annual Review of Pharmacology and Toxicology*.

[3] Guerra L. C., Del Carmen Fernández Moreno M., Chozas J. M. L., Hernández M. D. J. (2008). Statins in stroke prevention: what an internist should know. *European Journal of Internal Medicine*.

[4] Nassief A., Marsh J. D. (2008). Statin therapy for stroke prevention. *Stroke*.

[5] Park D.-S., So H.-S., Lee J.-H. (2009). Simvastatin treatment induces morphology alterations and apoptosis in murine cochlear neuronal cells. *Acta Oto-Laryngologica*.

[6] Borghi C., Modugno G. C., Pirodda A. (2002). Possible role of HMG-CoA reductase inhibitors for the treatment of sudden sensorineural hearing loss (SSHL). *Medical Hypotheses*.

[7] Zipp F., Waiczies S., Aktas O. (2007). Impact of HMG-CoA reductase inhibition on brain pathology. *Trends in Pharmacological Sciences*.

[8] Strome M., Topf P., Vernick D. M. (1988). Hyperlipidemia in association with childhood sensorineural hearing loss. *Laryngoscope*.

[9] Suzuki K., Kaneko M., Murai K. (2000). Influence of serum lipids on auditory function. *Laryngoscope*.

[10] Kojima Y., Ito S., Furuya N. (2001). Hearing improvement after therapy for hyperlipidemia in patients with chronic-phase sudden deafness. *Annals of Otology, Rhinology and Laryngology*.

[11] Olzowy B., Canis M., Hempel J.-M., Mazurek B., Suckfüll M. (2007). Effect of atorvastatin on progression of sensorineural hearing loss and tinnitus in the elderly: results of a prospective, randomized, double-blind clinical trial. *Otology & Neurotology*.

[12] Chung S.-D., Chen C.-H., Hung S.-H., Lin H.-C., Wang L.-H. (2015). A population-based study on the association between statin use and sudden sensorineural hearing loss. *Otolaryngology—Head and Neck Surgery*.

[13] Guo Y., Zhang C., Du X., Nair U., Yoo T.-J. (2005). Morphological and functional alterations of the cochlea in apolipoprotein E gene deficient mice. *Hearing Research*.

[14] Zhu S., Du X., Cai Q. (2008). Impaired stria vascularis in the inner ear of apolipoprotein E gene knockout mice. *ORL*.

[15] Cai Q., Du X., Zhou B. (2009). Effects of simvastatin on plasma lipoproteins and hearing loss in apolipoprotein E gene-deficient mice. *Oto-Rhino-Laryngology and Its Related Specialties*.

[16] Syka J., Ouda L., Nachtigal P., Solichová D., Semecký V. (2007). Atorvastatin slows down the deterioration of inner ear function with age in mice. *Neuroscience Letters*.

[17] Brand Y., Setz C., Levano S. (2011). Simvastatin protects auditory hair cells from gentamicin-induced toxicity and activates Akt signaling in vitro. *BMC Neuroscience*.

[18] Glutz A., Leitmeyer K., Setz C., Brand Y., Bodmer D. (2015). Metformin protects auditory hair cells from gentamicin-induced toxicity in vitro. *Audiology and Neurotology*.

[19] Leitmeyer K., Glutz A., Radojevic V. (2015). Inhibition of mTOR by rapamycin results in auditory hair cell damage and decreased spiral ganglion neuron outgrowth and neurite formation in vitro. *BioMed Research International*.

[20] Brand Y., Sung M., Pak K. (2015). Neural cell adhesion molecule nrcam is expressed in the mammalian inner ear and modulates spiral ganglion neurite outgrowth in an in vitro alternate choice assay. *Journal of Molecular Neuroscience*.

[21] Brand Y., Radojevic V., Sung M. (2014). Role of somatostatin receptor-2 in gentamicin-induced auditory hair cell loss in the mammalian inner ear. *PLoS ONE*.

[22] Sung M., Wei E., Chavez E. (2014). Inhibition of MMP-2 but not MMP-9 influences inner ear spiral ganglion neurons in vitro. *Cellular and Molecular Neurobiology*.

[23] Brand Y., Sung M., Chavez E. (2013). Neural cell adhesion molecule L1 modulates type i but not type II inner ear spiral ganglion neurite outgrowth in an in vitro alternate choice assay. *Journal of Molecular Neuroscience*.

[24] Van de Water T. R., Ruben R. J. (1971). Organ culture of the mammalian inner ear. *Acta Oto-Laryngologica*.

[25] Bogman K., Peyer A.-K., Török M., Küsters E., Drewe J. (2001). HMG-CoA reductase inhibitors and P-glycoprotein modulation. *British Journal of Pharmacology*.

[26] Duke R. C., Ojcius D. M., Young J. D. (1996). Cell suicide in health and disease. *Scientific American*.

[27] Payne C. M., Bernstein C., Bernstein H. (1995). Apoptosis overview emphasizing the role of oxidative stress, DNA damage and signal-transduction pathways. *Leukemia & Lymphoma*.

[28] Aletsee C., Beros A., Mullen L. (2001). Ras/MEK but not p38 signaling mediates NT-3-induced neurite extension from spiral ganglion neurons. *Journal of the Association for Research in Otolaryngology*.

[29] Chang N.-C., Yu M.-L., Ho K.-Y., Ho C.-K. (2007). Hyperlipidemia in noise-induced hearing loss. *Otolaryngology—Head and Neck Surgery*.

[30] Mortensen S. A., Leth A., Agner E., Ronde M. (1997). Dose-related decrease of serum coenzyme Q10 during treatment with HMG-CoA reductase inhibitors. *Molecular Aspects of Medicine*.

[31] Salami A., Mora R., Dellepiane M. (2010). Water-soluble coenzyme Q10 formulation (Q-TER®) in the treatment of presbycusis. *Acta Oto-Laryngologica*.

[32] Reid M. A., Flores-Otero J., Davis R. L. (2004). Firing patterns of type II spiral ganglion neurons in vitro. *The Journal of Neuroscience*.

[33] Henley C. M., Owings M. H., Stagner B. B., Martin G. K., Lonsbury-Martin B. L. (1990). Postnatal development of 2f1-f2 otoacoustic emissions in pigmented rat. *Hearing Research*.

[34] Rybak L. P., Whitworth C., Scott V. (1992). Development of endocochlear potential and compound action potential in the rat. *Hearing Research*.

[35] Ernfors P., Van de Water T., Loring J., Jaenisch R. (1995). Complementary roles of BDNF and NT-3 in vestibular and auditory development. *Neuron*.

[36] Echteler S. M., Nofsinger Y. C. (2000). Development of ganglion cell topography in the postnatal cochlea. *Journal of Comparative Neurology*.

